# Sensorimotor Integration Can Enhance Auditory Perception

**DOI:** 10.1038/s41598-020-58447-z

**Published:** 2020-01-30

**Authors:** John C. Myers, Jeffrey R. Mock, Edward J. Golob

**Affiliations:** Department of Psychology, University of Texas, San Antonio, USA

**Keywords:** Auditory system, Sensorimotor processing

## Abstract

Whenever we move, speak, or play musical instruments, our actions generate auditory sensory input. The sensory consequences of our actions are thought to be predicted via sensorimotor integration, which involves anatomical and functional links between auditory and motor brain regions. The physiological connections are relatively well established, but less is known about how sensorimotor integration affects auditory perception. The sensory attenuation hypothesis suggests that the perceived loudness of self-generated sounds is attenuated to help distinguish self-generated sounds from ambient sounds. Sensory attenuation would work for louder ambient sounds, but could lead to less accurate perception if the ambient sounds were quieter. We hypothesize that a key function of sensorimotor integration is the facilitated processing of self-generated sounds, leading to more accurate perception under most conditions. The sensory attenuation hypothesis predicts better performance for higher but not lower intensity comparisons, whereas sensory facilitation predicts improved perception regardless of comparison sound intensity. A series of experiments tested these hypotheses, with results supporting the enhancement hypothesis. Overall, people were more accurate at comparing the loudness of two sounds when making one of the sounds themselves. We propose that the brain selectively modulates the perception of self-generated sounds to enhance representations of action consequences.

## Introduction

Many of our actions have auditory consequences, such as hearing our own speech or playing musical instruments. Predicting the auditory consequences of our actions is important for motor control, and there is evidence that sensorimotor networks in the brain generate these predictions^1,2^. Predictions about features of action-related sounds (e.g., frequency or amplitude) are conveyed from motor to auditory networks to help coordinate movements and correct errors^3–6^. The underlying cognitive process of sensorimotor integration is thought to be subtractive, where the brain dynamically compares predictions about self-generated sounds to the actual consequences of the actions^5,7^. This comparison is also thought to be important for establishing the sense of agency in human actions, which is a core feature of consciousness^8,9^.

In this study we asked how sensorimotor integration affects the accuracy of auditory perception. We use term “active sounds” to refer to sounds that are generated by the participant. Active sounds are always a consequence of the participant’s actions. By contrast, “passive sounds” are presented to participants and are never a direct consequence of their actions. Researchers typically study the processing of active vs. passive sounds by having people speak or press buttons to generate sounds and then asking them questions about their perceptions (e.g., ‘Which sound was louder?’).

The neural basis of sensorimotor integration has been examined during spontaneous speech in humans and non-human primates. Electroencephalography (EEG), magnetoencephalography (MEG), and electrocorticography (eCoG) studies in humans consistently find smaller evoked responses to active sounds^10–14^. Similarly, when marmoset monkeys actively generate vocal calls, the mean firing rate of most neurons (~80%) in the auditory cortex is reduced, compared to when listening to passive playback of the same sound^12,13^. However, not all of the cells reduce their responsiveness, as about (~20%) have shown the opposite response, firing faster to the active sounds. The functional significance of the brain having different responses to active vs. passive sounds is unclear. One hypothesis is that the reduced firing rates of auditory cortical neurons during active sounds reflects the subtractive process that attenuates the perceived loudness of the auditory feedback^14,15^. However, the hypothesis that comparing predicted vs. actual sounds causes sensory attenuation does not fully account for the neurons that increase their response to active sounds. Possibly, the differential responses between neuronal populations might indicate a special case of predictive coding, which is a broader aspect of cognition involving predictions that the brain makes about the world (e.g., sensory predictions)^16–18^. Indeed, similar neural mechanisms might underlie both sensory and sensorimotor prediction, because even in the absence of movement, the premotor cortex has been shown to increase activity when participants try to predict audio/visual sequences^19^.

Several behavioral studies of auditory perception and sensorimotor integration have reported attenuated loudness perception for active sounds, leading to speculation that loudness attenuation might be a way to distinguish sensory inputs due to one’s own actions from other sources^20,21^. In previous studies, when participants were asked to judge which of two successive sounds was louder, the active sounds were generally interpreted as being slightly quieter than passively delivered sounds. This conclusion was largely based on psychophysical measures of the point of subjective equality (PSE)^5,21,22^. In the context of perceiving loudness, the PSE is the decibel value where the loudness of two stimuli are indistinguishable to the participant (i.e., 50% discriminability). For an ideal sensory observer, the mean dB difference would be 0. When actively triggering a sound, the PSE has been shown to be slightly less vs. a passive sound by less than 1 dB)^22^. Another quantification method involves measuring the accuracy of perceptual judgments relative to “ground truth” dB levels. Standard and test tones are presented in each trial, and within a block of trials the level of the test tone is always either above or below the standard dB level, and an observer's loudness judgment on each trial is either correct or incorrect. A value of accuracy, such as 75% which is intermediate to random and perfect performance with two choices, is then used to define a discrimination threshold. In this study, we took this approach to test whether the objective accuracy of auditory perception is affected by active vs. passive conditions.

A key limitation of the previous research is that stimulus order was not counterbalanced^5,22,23^. For these studies, on active trials the first sound was always active and the second sound was always passive. Prior studies have established that perceptual judgments can be influenced by stimulus order^24–26^, and thus one goal of the current study was to control for stimulus order. Another complication in the literature is that participants can be better at detecting active vs. passive sounds^27^. This suggests that, for very quiet sounds at the limits of detectability, active sounds are perceived to be louder, not quieter, than passive sounds. Taken together, the above observations show that loudness perception can differ between active and passively generated sounds. However, the specific patterns of results vary, and here are important methodological issues such as order effects that need to be addressed, which leaves the functional significance of the auditory-motor integrationunclear. The rationale for the current study is to directly test whether sensorimotor integration globally attenuates the perceived loudness of active sounds, or whether sensorimotor predictions can modulate perception in order to make more accurate judgments about the world. The purpose of sensorimotor integration is likely to modify sensory processing for action-related functions^3,14,28,29^.

There are theoretical reasons to question whether loudness attenuation can be used to distinguish sounds caused by one’s actions vs. other sources. First, using loudness to determine agency requires a precise representation of the expected loudness in memory; yet loudness matching based on memory is known to be imprecise^30^. Substantial noise is present not only in memory, but also in sound production, sound perception, and the environment. For example, the speech sounds of a repeated word are not acoustically identical^29^. Perceptual judgments of a given stimulus are also variable, which is the reason that experimental studies, including this one, average behavioral responses over many trials^31^. The perception of sensory feedback can also be masked by other sounds in the environment^32^. These four sources of noise (memory, motor output, sensory input, environment) are reasons to question whether humans can truly make such fine-grained loudness judgments that are reliable enough to distinguish sensory input from one’s actions vs. other sources. At the very least, more acoustic features, such as sound frequency may play a role in establishing agency.

**Figure 1:**
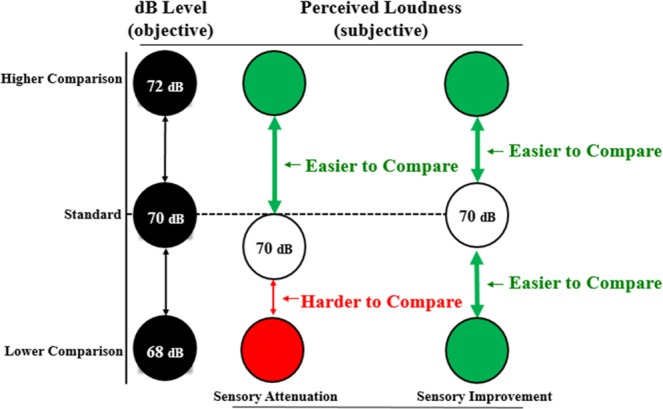
Model of how auditory-motor prediction would affect perception under two competing hypotheses. In this example the left column illustrates objective levels of a standard sound and comparison sounds that presented 2 dB above and below the 70 dB level of the standard. The middle column shows how ‘sensory attenutation’ would predicts that the perceived loudness of an active sound is attenuated, which should make it easier to judge loudness relative to a higher volume comparison sound. Conversely, it should be harder to compare loudness with the lower comparison sound. We propose that active sounds benefit from sensorimotor processing which should improve loudness judgments regardless of whether the comparison sound level is above or below the standard.

This study is comprised of three sound level discrimination experiments and one auditory detection experiment. In the discrimination task, two sounds are played in a row, and participants decide which one is louder. If perceived loudness is attenuated for active sounds, then subjects should be *more* sensitive to loudness differences when active sounds are less intense than the comparison sound (i.e., lower decibel level). Perceiving active sounds as quieter would exaggerate the perceptual difference between self vs. louder tones (see Fig. 1). Conversely, subjects should be *less* sensitive to loudness differences when active sounds are more intense than the comparison sounds. We hypothesize that sensorimotor integration will improve intensity discrimination performance regardless of which sound (active vs. passive) has the objectively higher sound level. Intensity discrimination can also be effected by expectations related to the sound level^33^. Thus, Experiment 1 included a between-subjects design to test whether active sounds are perceived differently when participants expect the passive sounds to be lower vs. higher intensity (±1–5 dB). In Experiments 2–3, intensity level direction was included as a within-subjects factor to test whether active sounds (always 70 dB) were perceived as louder or softer than passive sounds that were objectively ±2 dB away from the active sound (68 dB or 72 dB). The ±2 dB range was chosen because the accuracy of the perceptual judgments was near 75% in Experiment 1, equidistant from chance-level (50%) and perfect performance (100%). The role of sound feature expectation was examined by comparing loudness judgments of frequencies that, on the basis of previous training, were expected (75%) vs. unexpected (25%) frequencies. Note that the expectation about upcoming events and attention are inter-related, but can be experimentally distinguished^34^. Lastly, in Experiment 4 we hypothesized that low-intensity sounds would be more readily detected if they were self-generated and also matched the expected frequency.

## Methods and Results

### Experiment 1 methods

#### Experimental design

The purpose of Experiment 1 was to test whether sensorimotor integration improves sound intensity discrimination as a function of the predicted frequency and intensity. Experiment 1 consisted of a motor condition (*active*) and a non-motor control condition (*passive*). Participants first learned to associate pressing a button with pure tone auditory feedback (acquisition phase). Next, participants were asked to compare the loudness of the feedback to comparison tones of different intensities, using a two-alternative forced choice task. In the *active* condition, participants pressed a button to generate the standard reference tone, and comparison tones were always computer generated. In the *passive* condition the onset of both the standard reference and comparison tones were computer-generated.

#### Participants

University students (N = 42; female/male = 30/12; age, 21.26 ± 0.79 years; one left-handed) received course credit for participation. Pure tone thresholds were tested from 500 to 8000 Hz with an audiometer (Maico, Eden Prairie, MN, USA), and all participants were within normal hearing limits. All participants reported normal neurological and psychiatric health. Each participant signed an informed consent form in order to participate, and all experimental procedures were performed in accordance with a protocol approved by the University of Texas, San Antonio Institutional Review Board, consistent with the Declaration of Helsinki.

#### Procedure and stimuli

Participants were seated in an audiometric room in front of a computer monitor and keyboard with a pair of headphones (Audio-Technica, ATH-M20). Visual instructions were provided via computer monitor.

#### Acquisition phase

**Figure 2:**
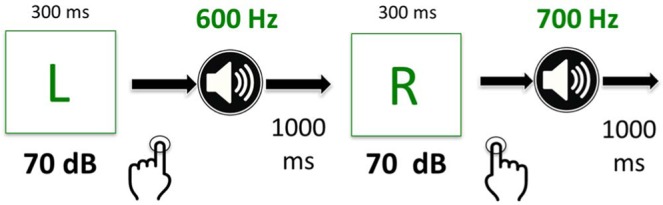
Experiments 1–3: Acquisition Phase Procedure. Participants pressed buttons to generate pure tones in response to visual cues (green ‘L’ or ‘R’) for 200 trials. Visual cues were presented for 300 ms to indicate which index finger to use to generate the feedback (left or right). For 50% of subjects, the left-key press generated a 600 Hz tone and the right-key press generated a 700 Hz tone (counterbalanced across all participants).

The acquisition phase is a training routine designed to evoke sensorimotor prediction by pairing a simple motor command (e.g., pressing a button) with a predictable sensory consequence (e.g., pure tone or somatosensory feedback)^1,5,22,35,36^. In Experiment 1, participants learned the association between a button press (left vs. right index finger) and a pure tone (600 Hz or 700 Hz, 250 ms, 10 ms rise/fall time, 70 dB SPL, (200 trials, ~3.5 sec/trial). In each trial, a visual cue (green ‘L’ or ‘R’, 300 ms) told the participants to push a key on a standard keyboard with either their left (‘ ~’ button) or right (‘+’ button) index finger to generate the onset of the auditory feedback (50 ms) (see Fig. 2). For 50% of subjects, the left-key press generated a 600 Hz *standard* tone and the right-key press generated a 700 Hz standard pure tone. The inter-trial-interval (ITI) was 1000 ms. Standard tone-to-key mapping was counterbalanced across all participants. We assigned distinct tone frequencies to each button in order to encourage the sense of agency and promote sensorimotor prediction during the task. Following the acquisition phase, a two-alternative forced choice (2AFC) procedure was used to measure the effects of sensorimotor integration on intensity discrimination.

#### Two-alternative forced choice task

**Figure 3:**
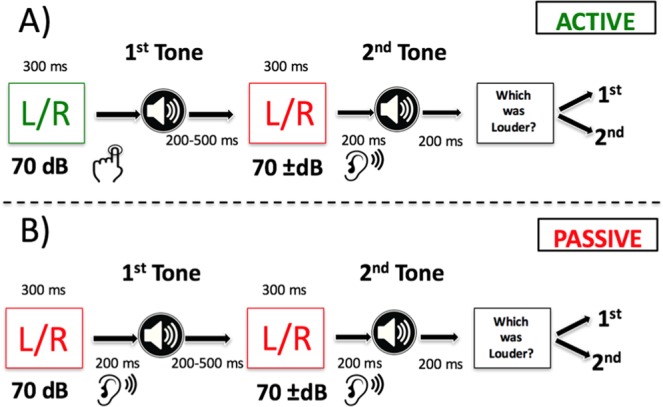
Experiments 1–3: Two-alternative Forced Choice Procedure. For each trial participants were presented with two consecutive tones and instructed to choose the louder tone. In the *active* condition (**A**) participants self-generated one of the two tones (always the standard tone at 70 dB) with a button press. In the *passive* condition (**B**) neither tone was produced by the participant. For 25% of trials, incongruent tones were presented that were originally mapped to the opposite visual cue and button press during the acquisition phase.

The test phase consisted of a 2AFC paradigm in which a standard reference tone (always 70 dB SPL) and a variable intensity comparison tone were presented consecutively in each trial. Half of the participants (n = 21) were presented with 5 *lower* intensity comparison tones (65, 66, 67, 68, or 69 vs. 70 dB), and the other half (n = 21) were presented with 5 *higher* intensity comparison tones (70 dB vs. 71, 72, 73, 74, or 75 dB). In the *active* condition, participants pressed a button to generate the standard tone in response to visual cues (green ‘L’ or ‘R’ for 300 ms). Tones were presented to participants 50 ms after pressing the button. In the *passive* condition, both the standard and comparison tones were externally-generated and linked with comparable visual cues to maximize predictability (red ‘L’ or ‘R’ for 300 ms). In both conditions, the standard tone was either preceded or followed by a ±1–5 dB externally-generated comparison tone (inter-stimulus interval (ISI) = 700–1000 ms). The time between the offset of the first sound and onset of the second sound, randomly varied between 700 ms, 850 ms, and 1000 ms (p = 0.33/inter-stimulus interval). The slight variability across trials was included to keep participants engaged in the task. Notably, when the second sound was the active sound (i.e., self-generated), the ISI was ~860 ms longer than when the first sound was active (i.e., 700/850/1000 ms +860 ms). This ISI discrepancy occurred because participants were given an unfixed amount of time to generate the sound. At the end of each trial, participants were asked, ‘Which tone was louder?’ The next trial began 1300 ms after subjects made their loudness judgment (see Fig. 3).

To test the importance of hearing the expected frequency to auditory motor prediction, 25% of the trials contained *incongruent* frequencies that, in the acquisition phase, were mapped to the opposite response. All trials were counterbalanced for stimulus order (standard tone 1^st^ vs. 2^nd^) (see Fig. 3), and the two tones always had the same frequency. Active/passive trials were presented in separate, alternating blocks consisting of 56 trials (560 total trials; 5 blocks/condition). The left vs. right cue for each trial was always random (50% probability).

#### Data analysis

Psychometric (logistic) functions were fit to each subject’s percent correct discrimination as a function of standard vs. comparison intensity (±1–5 dB) data using a maximum likelihood procedure. Psychometric functions were analyzed for each intensity level direction (between-subjects factor: higher dB or lower dB), condition (within-subjects factor: active, passive), frequency (within-subjects factor: congruent, incongruent), and stimulus order (within-subjects factor: standard tone 1^st^ or 2^nd^). Intensity discrimination *slope* was defined as the estimated percent increase in performance for every 1-dB change in intensity. Each measure provides important information about how perception is affected by the motor task. Intensity discrimination thresholds and slopes were assessed using a 2 (intensity level direction: ±1–5 dB) × 2 (condition: active vs. passive) × 2 (frequency: congruent vs. incongruent) × 2 (stimulus order: standard tone first or second) mixed analysis of variance (ANOVA). Therefore, each subject had eight threshold and slope values, one for each condition (active vs. passive). Intensity discrimination *threshold* was defined as the point on the psychometric function corresponding to 75% correct discrimination, halfway between chance (50%) and perfect performance^24,37^. Effect sizes were computed using partial eta squared (*η*_*p*_^2^). In Experiment 1, intensity level direction was included as a between-subjects factor to separately evaluate the effect of consistent louder vs. quieter comparison tones on perceptual sensitivity. Making judgments on variable amplitude comparisons might implicitly impact perceptual judgments. Later, Experiments 2–3 include intensity level direction as a within-subjects factor.

### Experiment 1 results

**Figure 4:**
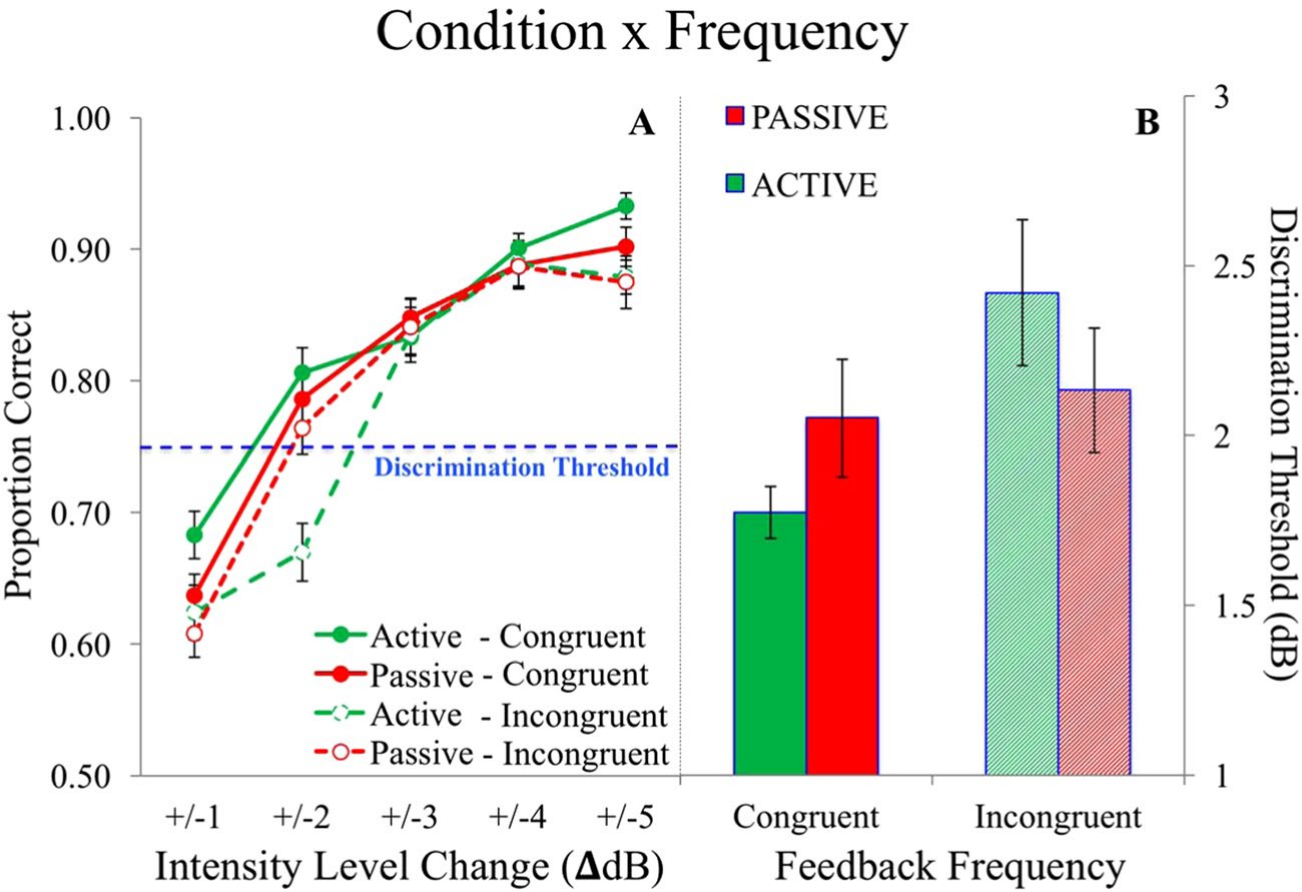
Experiment 1 - Effect of motor commands and feedback frequency on intensity discrimination across each intensity level. In the active condition, sensitivity diminished when actively generating incongruent sounds (**A**: *p* < 0.010). Discrimination thresholds were highest for self-generated, incongruent feedback (**B**: *p* < 0.050). Error bars reflect standard error of the mean.

**Figure 5:**
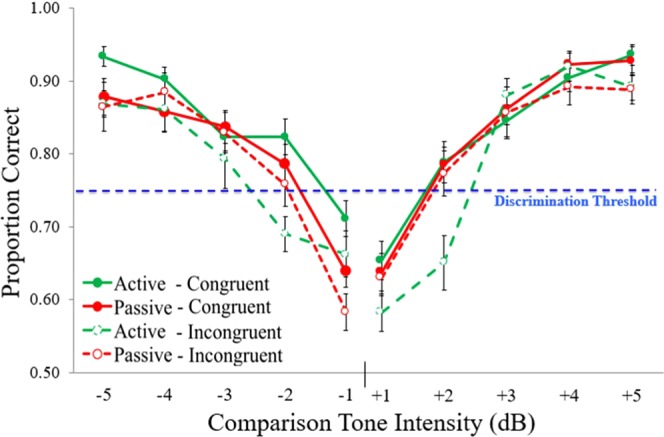
Experiment 1. Condition × congruence effects between subjects, where comparison tone intensities were either higher or lower than the 70 dB standard. In both the up and down directions, discrimination thresholds were higher when generating incongruent frequencies, which suggests that perception is biased towards predicted action effects (*p* < 0.010). Error bars reflect standard error of the mean.

A 2 (intensity level direction) × 2 (condition) × 2 (frequency) × 2 (stimulus order) ANOVA indicated an interaction of condition × frequency (*F*_(1,40)_ 9.79, *p* < 0.010, *η*_*p*_^2^ = 0.20; Fig. 4). In the active condition, thresholds were lower for congruent and higher for incongruent frequencies (*F*_(1,40)_ = 25.32, *p* < 0.001, *η*_*p*_^2^ = 0.39). Discrimination thresholds (intensity level needed to get 75% accuracy) decreased when self-generated frequencies were congruent (1.771 ± 0.08 dB to get 75% accuracy), but increased when frequencies were incongruent (2.27 ± 0.10 dB to get 75% accuracy) (Fig. 4A). In the passive condition, congruent vs. incongruent frequencies did not affect thresholds (*p* = 0.40). The mostly symmetrical effect for higher vs. lower intensity is shown in Fig. 5. Stimulus order (standard 1^st^ vs. 2^nd^) had a trending main effect on discrimination thresholds, but the effect was not significant (*p* = 0.091).

For the slope measure there were no significant effects involving active vs. passive conditions. However, there was a main effect of frequency, showing a steeper slope for the congruent frequency in both conditions (*F*_(1,40)_ 6.10, *p* = 0.018, *η*_*p*_^2^ = 0.13) (see Fig. 5). There was a trend for a condition × intensity level interaction, where lower dB comparison tones had steeper slopes vs. when comparison tones had higher levels than the standard (*p* = 0.070, *η*_*p*_^2^ = 0.08).

The results of Experiment 1 provide evidence that actively generating a tone affects loudness threshold judgments, with greater perceptual acuity for expected vs. unexpected tones. Discrimination thresholds during passive listening were in-between those of the active expected and unexpected tones, and were comparable for expected and unexpected passive tones. The slope results show that the steepness of the relation between intensity differences and loudness judgments was not influenced by active vs. passive stimulus delivery. Instead, overall sensitivity to a difference being present was greatest for active-expected tones, intermediate for passive tones, and least for active-unexpected tones.

### Experiment 2 methods

#### Experimental design

The purpose of Experiment 2 was to test the effect of auditory-motor prediction on perceptual *accuracy* at intensities above and below the self-generated intensity (70 ± 2 dB). In Experiment 1, the intensity level direction of the comparison tone was a between-subjects factor. Half of the participants were presented with lower volume comparison tones and the other half compared the 70 dB standard to higher volume tones. Experiment 2 was a simplified intensity discrimination task consisting of only 68 & 72 dB comparison tones that were tested within-subjects. If auditory-motor predictions are more accurate relative to passive predictions, then discrimination accuracy should be higher in an environment/context of variable intensity and frequency. Our central hypothesis was that accuracy would be greater in the active vs. passive condition. We also hypothesized that congruent vs. incongruent frequencies might affect perception differently when a bidirectional range of intensities is presented vs. unidirectional as in Experiment 1.

Just as in Experiment 1, Experiment 2 consisted of an active condition and a non-motor passive condition. Subjects pressed buttons to generate standard tones (70 dB) in an identical acquisition phase, followed by a similar 2AFC intensity discrimination task. The independent variables were condition (active vs. passive), frequency (congruent (75% of trials) vs. incongruent (25% of trials)), stimulus order (standard tone 1^st^ vs. 2^nd^), and intensity (−2 dB vs. +2 dB). Recall that when the subjects actively produced a tone it was always the standard, thus the order of actively produced tones was also counterbalanced across trials. Subjects were tested with both −2 dB and +2 dB comparison tones in Experiment 2, as opposed to −1 to −5 dB or +1 to +5 dB in Experiment 1. The active and passive trials were presented one after the other in interleaving blocks, consisting of ~50 trials per block (592 total trials; 6 blocks/condition). The number of trials was equivalent across active and passive conditions.

#### Participants

Participants were a new set of university students (n = 24; female/male = 20/4; age = 18.23 + 0.12 years; all right-handed) who received course credit for participation.

#### Data analysis

Intensity discrimination accuracy was used to measure perceptual sensitivity as a function of auditory-motor prediction. The data were then re-analyzed by calculating sensitivity (d-prime (d’)) using signal detection theory^38^, the statistical findings did not differ from those reported below (see Supplementary Materials). For simplicity’s sake, only discrimination accuracy (% correct) is presented below. Discrimination accuracy effects were quantified by a 2 (condition: active vs. passive) × 2 (frequency: congruent vs. incongruent) × 2 (stimulus order: standard tone 1^st^ or 2^nd^) × 2 (intensity: −2 dB or +2 dB) analysis of variance (ANOVA) test.

### Experiment 2 Results

**Figure 6:**
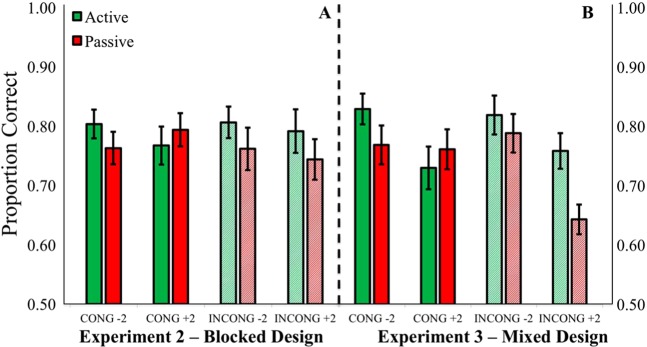
Experiments 2 and 3 – Sensorimotor integration improved intensity discrimination under most conditions (*p* < 0.001). The main exception was that self-generated congruent tones were harder to compare to higher intensity externally generated tones (i.e., CONG +2: self −70 dB vs. other −72 dB). Error bars reflect standard error of the mean.

Discrimination accuracy showed a main effect of condition (*F*_(1,23)_ = 4.64, *p* = 0.042, *η*_*p*_^2^ = 0.17)); accuracy was higher in the active vs. passive condition (active M = 0.79, SE = 0.03, passive M = 0.76, SE = 0.03). Accuracy was greater in the active condition in 67% of subjects, with a mean perceptual benefit of 2.7 ± 0.8%. A three-way condition × frequency × intensity interaction was observed, *F*_(1,23)_ = 7.39, *p* = 0.012, *η*_*p*_^2^ = 0.24. The interaction indicated that under most conditions, participants showed greater perceptual accuracy in the active condition, except when self-generating congruent frequency tones that were lower intensity than the comparison tone (70 dB vs 72 dB) (see Fig. 6A).

**Figure 7:**
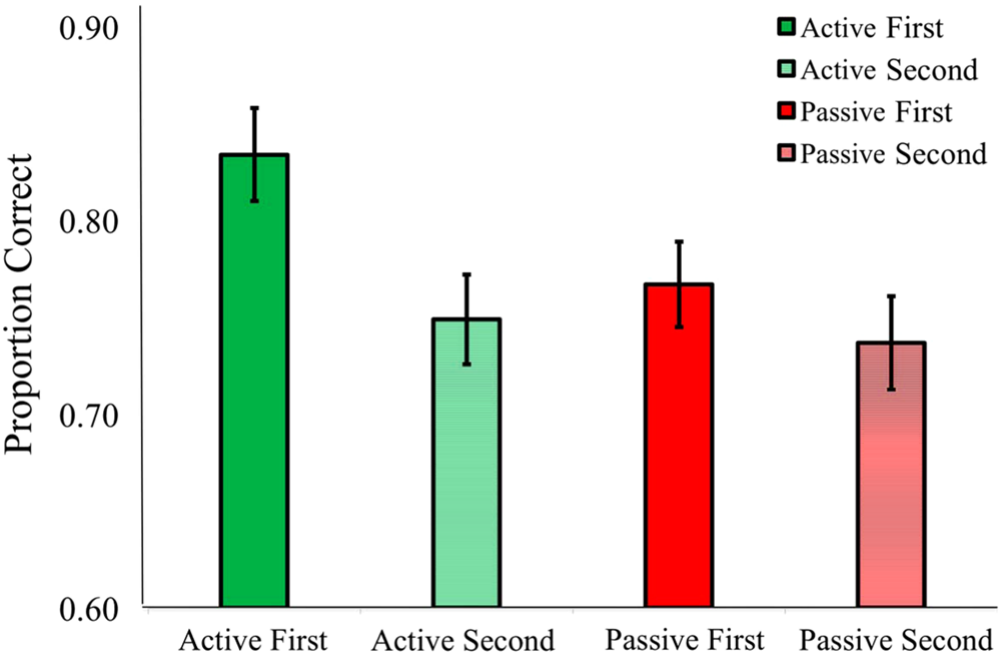
Condition × Stimulus Order. Across Experiments 2–3, there was a main effect of stimulus order, indicating that participants were better at intensity discrimination when the standard tone was presented first (*p* < 0.001). The interaction suggest that auditory-motor prediction can improve mental representations of self-generated sounds for better comparison with subsequent stimuli. Error bars reflect standard error of the mean.

There was also a main effect of stimulus order (*p* = 0.006, *η*_*p*_^2^ = 0.29), indicating better performance when the standard tone was presented first. Beyond the main effect, a strong condition × stimulus order interaction indicated that performance in the active condition improved when the self-generated standard tone was presented first (M = 0.83, SE = 0.03) vs. second (M = 0.75, SE = 0.03) (*p* = 0.002, *η*_*p*_^2^ = 0.35) (see Fig. 7). The passive condition was unaffected by stimulus order (*p* = 0.58). The results of Experiment 2 suggest that sensorimotor prediction improves intensity discrimination accuracy under most conditions, and is greater when the standard tone is presented first.

### Experiment 3 methods

#### Experimental design

The purpose of Experiment 3 was to replicate the findings of Experiment 2 in a more dynamic context. To test whether the increased accuracy was due to active and passive trials being in separate blocks, Experiment 3 consisted of an identical design to Experiment 2 with one added caveat. Instead of presenting active and passive trials in separate blocks, the trials of each condition were interleaved. Thus the nature of each upcoming trial was unpredictable, and could be either active or passive. The active and passive trial types were presented randomly, consisting of ~50 trials per block (592 total trials; 6 blocks/condition).

#### Participants

A new group of university students were recruited (n = 24; female/male = 14/10; age, 19.46 ± 0.68 years; six left-handed, one ambidextrous), and they received course credit for participation.

#### Data analysis

Intensity discrimination accuracy effects were again quantified by a 2 (condition: active vs. passive) × 2 (frequency: congruent vs. incongruent) × 2 (stimulus order: standard tone 1^st^ or 2^nd^) × 2 (intensity: −2 dB or +2 dB) analysis of variance (ANOVA) test.

### Experiment 3 results

Consistent with the results of Experiment 2, discrimination accuracy had a main effect of condition, *F*_(1,23)_ = 23.92, *p* < 0.001, *η*_*p*_^2^ = 0.51, and was better in the active (M = 0.79, SE = 0.02) vs. passive condition (M = 0.74, SE = 0.03). The active condition had the greatest accuracy in 75% of subjects, with the average perceptual benefit equaling 5.7 ± 1.1%. There was a condition × frequency interaction (*p* < 0.001, *η*_*p*_^2^ = 0.40), showing that the active condition was unhindered by incongruent frequencies (~1% difference), while performance worsened in the passive condition for incongruent frequencies (~7% difference). A similar three-way condition × frequency × intensity interaction was observed, indicating greater perceptual accuracy in the active condition, except when self-generating lower intensity congruent frequencies, *F*_(1,23)_ = 16.67, *p* < 0.001, *η*_*p*_^2^ = 0.42 (see Fig. 6B).

There was a strong main effect of intensity (*p* = 0.001, *η*_*p*_^2^ = 0.62), showing that subjects were consistently better at making loudness judgments when the comparison tone was 68 dB (M = 0.80, SE = 0.03) vs. 72 dB (M = 0.73, SE = 0.02) (see Fig. 6B). Stimulus order also presented with a robust main effect (*p* < 0.001, *η*_*p*_^2^ = 0.64), showing again that accuracy improved in both conditions when the standard tone was presented first (M = 0.80, SE = 0.20) vs. second (M = 0.74, SE = 0.02). An order × intensity interaction indicated that performance worsened in both conditions when the 72 dB comparison tones were presented before the 70 dB standard tones (*p* = 0.025, *η*_*p*_^2^ = 0.20). In accordance with Experiment 2, here we observed another condition × stimulus order interaction, suggesting that the benefits of presenting the standard tone first were greater in the active condition (*p* = 0.026, *η*_*p*_^2^ = 0.20). The standard tones may have attained an expectation benefit because they are presented on every trial as a reference point for comparison (see Fig. 7 for order effects across Experiments 2–3).

**Figure 8:**
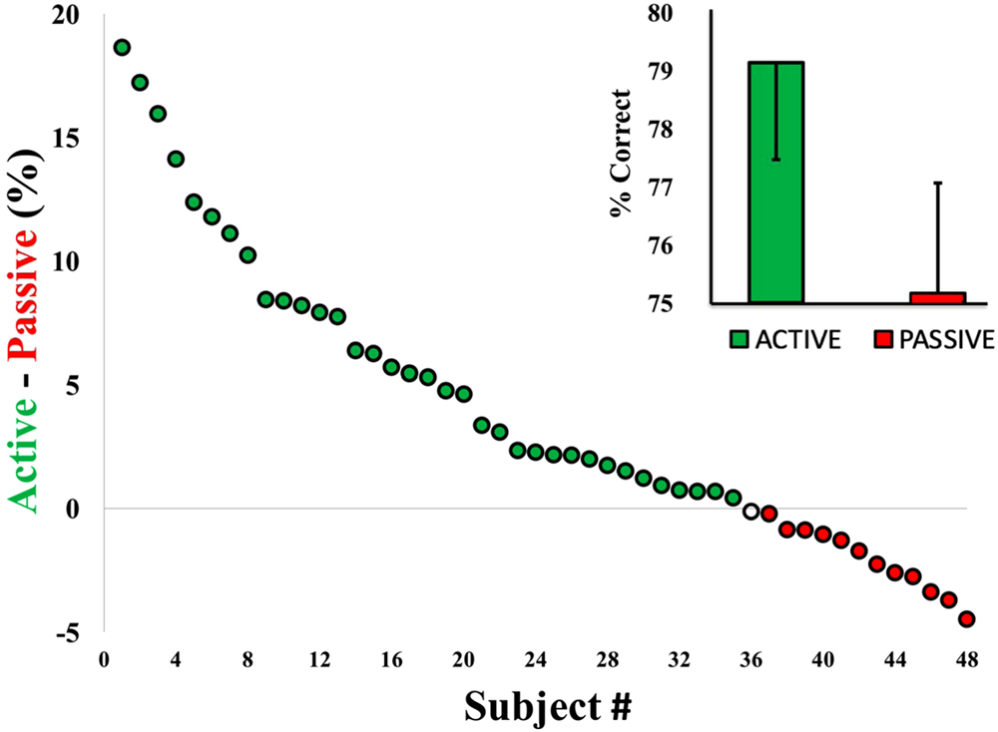
Experiments 2 and 3. Auditory-motor prediction improved intensity discrimination for most subjects in Experiments 2–3 (green circles). Red circles mark subjects who performed better in the passive condition. Results demonstrate substantial perceptual benefits for sensorimotor prediction that exceed any expectation benefits in the passive condition (*p* < 0.001). Error bars reflect standard error of the mean.

The results of Experiment 3 replicated the findings of Experiment 2 within a more variable context of a mixed design. Across Experiments 2 and 3, self-generating the standard tone had superior intensity discrimination vs. passive delivery in 75% of the participants. In some subjects, sensorimotor perception exceeded passive performance by nearly 20% (see Fig. 8).

### Experiment 4 methods

#### Experimental design

In Experiments 1–3 we used intensity discrimination tests to determine the effects of sensorimotor prediction on auditory perceptual acuity. In Experiment 4, we measured auditory *detection* by employing a similar paradigm which comprised an acquisition phase followed by an adaptive staircase procedure^39^. The purpose of Experiment 4 was to determine if sensorimotor prediction improves auditory stimulus detection as a function of congruent vs. incongruent frequencies.

#### Participants

Participants were university students (N = 22; female/male ratio, 12:10; age, 20.64 ± 0.70 years; one left-handed), who received course credit for participation.

#### Acquisition phase

Participants learned to associate an index finger button press with ‘target’ tone feedback (600 Hz or 1000 Hz, 250 ms duration, 10 ms rise/fall time, 70 dB SPL). Each trial, a visual cue (green lettered ‘PRESS’, 300 ms) instructed participants to press a button to generate target congruent frequencies. If participants responded too slowly (reaction time >350 ms) then auditory feedback was altered to generate incongruent frequencies above or below the target (i.e., ±100 Hz). The incongruent feedback was designed to ensure that participants performed the single-button task attentively, thus forming a strong sensorimotor association. The task continued until a minimum of 90% of correct responses were reached (~200 trials, ~3.5 sec/trial). Each subject was tested in two frequency ranges (500–600–700 Hz & 900–1000–1100 Hz). In each range the middle frequency was the target tone, and the incongruent frequencies were ±100 Hz.

#### Detection test phase

In alternating trials, visual cues indicated when to press the button for the congruent target tone (active cue: green ‘PRESS’) vs. when to simply listen for the target (passive cue: red ‘LISTEN’). The target tones were presented at a starting 70 dB. Following each auditory stimulus, participants were asked if they perceived the sound by pressing a ‘Yes’ or ‘No’ button (‘left’ or ‘right’ arrow keys). If they reported ‘Yes’ to hearing the tone, the next tone would decrease intensity by variable step sizes (−10, −5, or −1 dB). If they reported ‘No’ to hearing the tone, the next stimulus would increase intensity (+5, +2.5, or +0.5 dB). Whenever participants reversed their responses from ‘Yes’ to ‘No’ or vice versa, the corresponding intensity value was recorded as a ‘reversal.’ Step size varied incrementally on each reversal. We used the average intensity after 12 reversals to estimate detection thresholds. After the initial detection thresholds for the congruent frequencies were estimated in both active and passive conditions, the two incongruent non-target frequencies (±100 Hz from targets) were introduced at intensities *equaling* the congruent threshold. Detection thresholds were tested in two frequency ranges for each subject (500, 600 target, 700 Hz; 900, 1000 target, 1100 Hz). To measure the consistency of detection thresholds for congruent frequencies, we continued to re-estimate the target detection thresholds by resetting the intensity back to 70 dB (≥12 additional reversals) until the thresholds for incongruent frequencies (25% of trials) were reached.

#### Data analysis

Congruent target thresholds were measured at least 5 times for each subject. The thresholds of 3 subjects were rejected on the basis of extreme variability across the five measures (i.e., more than ±3 standard deviations from the grand mean). The threshold data were then feature-scaled between 0–1 across frequency ranges for each subject, because auditory thresholds have been shown to vary across frequencies (500–1100 Hz)^40,41^. Detection thresholds were analyzed using a 2 (range: 500–700 Hz vs. 900–1100 Hz) × 2 (condition: active vs. passive) × 3 (frequency: target, +100 Hz, −100 Hz) ANOVA.

### Experiment 4 results

**Figure 9:**
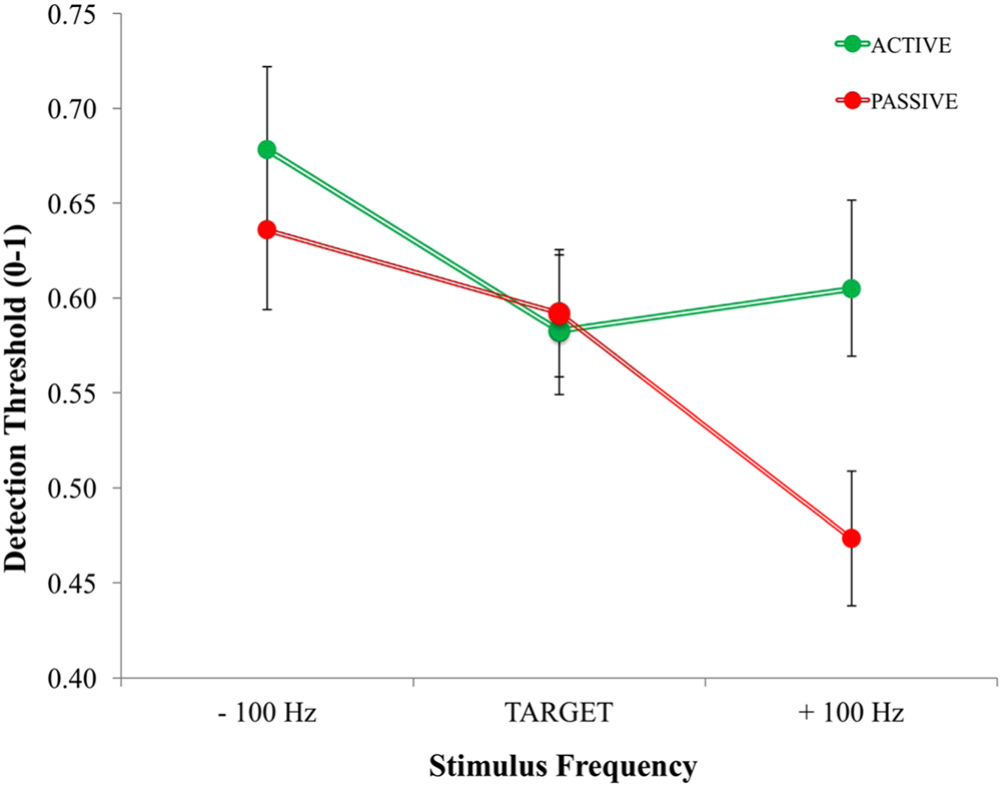
Effects of auditory-motor prediction × feedback frequency on detection thresholds. Target tone detection thresholds did not differ between active and passive conditions. However, mean detection thresholds for the less common (25%) non-target frequencies (−100 & +100 Hz) were nearly 2 dB *higher* in the active condition (*p* = 0.019). Error bars reflect standard error of the mean.

Target detection thresholds did not differ between active and passive conditions (*p* = 0.826), but there was a significant condition × frequency interaction, (*F*_(2,34)_ = 4.47, *p* = 0.019, *η*_*p*_^2^ = 0.21). The interaction showed that thresholds for active and passive conditions were comparable for congruent targets, but thresholds for incongruent tones were nearly 2 dB *higher* in the active condition (active M = 4.68, SE = 1.32, passive M = 2.69, SE = 1.94) (see Fig. 9). In both active and passive conditions, the detection thresholds were lower for the higher frequencies (*F*_(2,34)_ = 6.06, *p* = 0.006, *η*_*p*_^2^ = 0.263), which is a general property of the human auditory system^37^. However, as given by the interaction mentioned above, the detection thresholds for the higher frequency incongruent tones was greater in the active condition. The results of Experiment 4 provide evidence that sensorimotor integration could involve selective filters for the predicted frequency content of the auditory action consequences, especially when stimuli are more difficult to hear, at near-threshold intensity levels. Sensorimotor integration may maintain the detectability of target frequencies while decreasing sensitivity for non-targets^42^.

## Discussion

The primary aim of this study was to test whether actively producing a sound improves the accuracy of auditory perception. The secondary aim was to determine if there was any effect of expected vs. unexpected frequencies, which has important implications for attention control and the sense of agency. Theoretically, a key function of sensorimotor integration is to maximize action performance, especially for actions that have variable sensory consequences^4^. The results of Experiment 1 showed effects related to ‘self-’ vs. ‘other-’ sound production and whether the frequency of the sound was expected vs. unexpected. Discrimination thresholds were the lowest when self-generated sounds occurred at the expected (congruent) frequency and highest when the frequency of the self-generated sound was unexpected (incongruent) frequency. This pattern of frequency-specific facilitation was seen regardless of whether the self-generated tones were louder or softer than the comparison tones between subjects.

In Experiments 2 and 3, accuracy was tested in a context where the loudness judgments were made between sounds that were ±2 dB apart. We chose this ±2 dB range because that was the intensity level where performance was closest to the 75% discrimination threshold in Experiment 1. The results of Experiments 2–3 showed that ~75% of the participants made more accurate perceptual judgments in the active condition, regardless of the frequency of the self-generated output. Lastly, absolute detection thresholds in Experiment 4 did not differ for active vs. passively produced sounds at the expected frequency, but were somewhat higher in the active condition to unexpected frequency.

Overall, the findings in Experiments 1–3 showed a mostly symmetrical pattern where accuracy is greater when judging actively triggered sounds relative to passive presentation. The only exception was observed in both Experiment 2 and 3. The accuracy advantage for active sounds was not evident when the standard was at the expected frequency and the comparison tone was slightly louder. This finding was replicated, and future work would be needed to better understand its significance.

Although Experiments 1–3 were consistent on the main finding that accuracy was greater for active vs. passive tones, there was a difference with respect to congruent vs. incongruent frequencies. In experiment 1 the threshold for active congruent trials was lower than active incongruent trials, while congruent and incongruent trials had comparable results in Experiments 2 and 3. One possibility for the difference is that there was a greater range of levels (5 vs. 2 in Experiment 2 and 3) and discrimination difficulty (1 to 5 dB in Experiment 1 vs. 2 dB in Experiments 2 and 3). We speculate that the conditions in Experiment 1 may have encouraged an increased attention to the frequency of the tones (congruent vs. incongruent), especially in the active condition where participants generated one of the sounds themselves. The role of task difficulty in differences between congruent and incongruent sound may also apply to Experiment 4, where low-intensity detection thresholds were significantly higher for incongruent frequencies, but only in the active condition (see Fig. 9). In the future, more research could be done to directly examine the effect of discrimination conditions and task difficulty on sensorimotor integration in perception.

Previous work suggests that motor control includes both action programs and a forward model that predicts the sensory consequence of an action^14^. A subtractive comparison between actual and predicted sensory feedback is performed to fine tune motor control, and the reduced perceived loudness specified by the attenuation hypothesis is thought to be a byproduct of this subtraction^5,20,22^. Loudness attenuation is also thought to help distinguish sounds from one’s own actions from those generated by other sources^21^. Both of these concepts rely on the premise that action sound effects are generally perceived as quieter than objective reality.

The results from Experiments 1–3 did not support the attenuation model’s core idea that a self-generated sound is perceived to be quieter relative to when the same sound is triggered by other means. If the perceived loudness of actively generated standards was reduced then participants would have had better discrimination performance when the level of comparison sounds exceeded the standard, and worse performance when the level of comparison sounds was less than the standard. Instead, actively producing the sound had comparable perceptual benefits regardless of whether the comparison tone was more or less intense than the standard. Indeed, performance accuracy favoring the active condition was somewhat better when using quieter comparison tones.

Overall, the impact of active generation on loudness perception is subtle, but reliable, and is seen both within and between different study protocols. The subtle nature shows that the comparison of expected and actual sensory feedback in motor control does not substantially distort perception. As with other influences on auditory perception such as spatial biases by eye position^43^, choice of effectors^44^, and numerosity^45^. The perceptual benefits of actively producing the sound are comparable to the magnitude of just-noticeable-differences. This preserves accurate perception, and only by averaging over many trials are the subtle perceptual biases revealed.

### Stimulus order effects

A major difference between this study and prior work is that earlier studies using 2-alternative forced choice procedures always presented the self-generated sound first on each trial^5,22^. This was typically done because a different variable, such as a manipulation related to agency, was of primary interest and controlling for order would have added many more trials. Nonetheless, it is important to counterbalance stimulus order because it can influence neural responses, perceptual judgements, and lead to response bias^25,46,47^. In Experiments 1–3 stimulus order was counterbalanced, and in Experiments 2–3 order effects were specifically examined. There were large order effects due to greater accuracy in the active condition when the self-generated standard tone was presented first rather than second. When the active sound was second in the sequence, ISI was longer by ~860 ms due to longer participant reaction times (active 1^st^ ISI: 700–1000 ms; active 2^nd^ ISI: ~1560–1860 ms). The increased time between sounds may have diminished the perceptual benefits of sensorimotor integration down to passive levels. The findings suggest that in studies where active sounds could only occur on the first stimulus the difference between active and passive conditions on loudness perception might have been overestimated. However, a limitation of this study is that the average time between tones was longer when the active tone was presented second, which could have also influenced performance.

When comparing two successive sounds, the first sound serves as a referent that is maintained in echoic memory. The memory of that first tone is then used to make a loudness judgment when the second tone is presented. In Experiments 2–3, we found that the perceptual benefits of active sounds were mainly present when the first of the two sounds was self-generated. We suspect that the benefits of actively generating the first tone could be related to an improved precision or increased durability of the echoic memory trace for active sounds, possibly due to an attentional benefit. Echoic memory is fleeting, with progressive decay occurring over several seconds^30,48^. A more precise echoic representation of the first tone’s loudness, and/or greater resistance to decay over time, would lead to better performance when the memory of the first tone is compared to perception of the second tone. For passive sounds, we found no differences in accuracy related to stimulus order, suggesting that the order effects were not due to general stimulus factors such as refractory effects^46,49^.

Future work is needed to better equate the time between tones when the active tone is presented first vs. second. This would control for the likelihood of greater echoic memory decay when the active tone was presented second. It follows that one reason that the active vs. passive difference is smaller when the active tone is second could be that overall loudness discrimination is worse due to echoic memory decay. The results in Fig. 7 do not support this interpretation, because accuracy when active tones were presented second was about the same as with the two passively delivered tones that a shorter interval between tones. If actively generated tones do yield a benefit in terms of precision and/or resistance to decay in echoic memory, as suggested above, then another possibility is that these echoic memory benefits are less useful because subjects make their loudness judgment right after hearing the second tone.

Previous studies of auditory-motor integration focused almost exclusively on the point of subjective equality (PSE) where sensory attenuation was indicated by lower PSE values for active vs. passive conditions^5,9,21^. This study focused on measures of perceptual acuity (discrimination threshold and accuracy), rather than testing for shifts in the PSE. Even though there are similarities between perceptual acuity and the PSE, the differences are also relevant to interpreting the findings. Changes in the PSE indicate subjective effects of sensorimotor integration because the dB threshold for PSE is near 50% discriminability^22^. Changes in discrimination threshold is a more objective measure of perceptual acuity because the discrimination threshold is set to 75% accuracy, where subjects can reliably discriminate between the loudness of two sounds. Note again that the ±2 dB differences were examined in Experiments 2 and 3 were near the 75% threshold observed in Experiment 1. Future studies on sensorimotor integration and perception should control for stimulus order, as was done here, in tandem with examining a variety of perceptual measures to help reconcile mixed findings among studies.

We emphasize that from the perspective of a sensory system, the information corresponding to sensory feedback from an action is not a given because there is often more than one sound source in the environment at the same time. The sensory consequences of an action need to first be identified before being compared to the prediction of a forward model. We speculate that the small perceptual differences between active and passively generated sounds may reflect the selection of sensory input that is created by motor output. An alternative possibility is that perceptual differences for active vs. passive stimuli reflect a subsequent comparison between the selected sensory information and the forward motor model. The mechanisms of this selectivity are unclear, but may have similarities to biased competition models of attention, with the forward model providing the bias toward sensory features of the expected feedback^50,51^. Many studies in the visual modality, and to a lesser degree in audition, show that manipulations of attention can influence perception^32,52^. Human EEG data suggest that attention effects are present in a typical active vs. passive task, but are dissociable from motor control effects^10^. The results from Experiment 1 are broadly consistent with an expectation bias, but in Experiments 2 and 3 there were no substantial differences between the expected and unexpected frequencies. In addition, if the benefits on performance for active sounds are a special case of predictive coding then attending to and/or expecting the stimuli may not be necessary^53^. Clearly future work is needed to more fully test the effects of sensorimotor integration and the potential roles of expectation and attention.

As described above in the context of sources of noise, the impact of active generation on loudness perception is subtle, but reliable, and is seen both within and between different study protocols. The subtle nature shows that the comparison of expected and actual sensory feedback in motor control does not substantially distort perception. As with other influences on auditory perception such as spatial biases by eye position^43^, choice of effectors^44^, and numerosity^45^, the perceptual benefits of actively producing the sound are comparable to the magnitude of just-noticeable-differences. This preserves accurate perception, and only by averaging over many trials are the subtle biases revealed.

### Potential multisensory influences

In addition to self-generated movement, the active and passive conditions also differ by the presence vs. absence of tactile feedback from pressing a button. We speculate that the tactile feedback from button pressing could have influenced the results because loudness ratings increase with concurrent tactile stimulation^54,55^. However, if better performance in the active condition was only due to tactile feedback then condition would not have interacted with any other factor. Instead, condition interacted with stimulus type (Experiment 1), order (Experiments 2, 3), and the condition effect was absent for congruent trials with more intense comparison tones (Experiments 2, 3). We conclude that it is worth investigating the possibility of tactile influences in these types of tasks, but it cannot fully explain the present set of results.

The first three experiments measured discrimination of clearly audible sounds. Experiment 4 tested whether detection of near-threshold tones differed between active and passive presentation. At the expected frequency thresholds were nearly identical in the active and passive conditions. For the unexpected frequency thresholds were greater in the active condition, which suggests that unexpected frequencies were filtered in the active condition. Other studies that examined detection of signals in noise also support the idea of an attentional filter that is centered on an expected frequency^32,56^. In contrast, Reznik and colleagues found that detection thresholds were lower for actively delivered sounds vs. passive listening^27^. In the current study, we did not find lower detection thresholds for active sounds. Relevant differences between studies include having a mixed trials design here vs. a blocked design in Reznik and colleagues. The current study also included a passive condition that promoted expectations for frequency, which can dissociate the perceptual effects of expectation from motor control. We also note that using incongruent tones during the training phase might have influenced threshold testing vs. using novel tone frequencies. The rationale was that all of the sounds at testing would be familiar to the subjects, and thus less likely to elicit novelty-related activity that could, at least initially, interfere with threshold testing.

In everyday life, auditory-motor control enables us to speak fluidly and distinguish the sounds we make from the sounds generated by other sources. This study has strengthened the current understanding of action-effects on perception and increased our knowledge about how perception can be more accurate when the motor system is engaged. Faulty auditory-motor integration is associated with a variety of clinical problems, ranging from stuttering disfluency^57,58^ to auditory hallucinations common in schizophrenia (i.e., misattributions of self- vs. other auditory input)^59,60^. Understanding how the brain integrates sensory and motor information is essential to understanding consciousness, human behavior, and mental health. Even the sense of agency in our actions is thought to depend on sensorimotor interactions^22,61–63^. Therefore, sensorimotor integration will continue to drive further research.

## Supplementary information

Dataset 1.
